# Packaging of Fresh Sliced Mushrooms with Essential Oils Vapours: A New Technology for Maintaining Quality and Extending Shelf Life

**DOI:** 10.3390/foods10061196

**Published:** 2021-05-26

**Authors:** Antonio López-Gómez, María Ros-Chumillas, Alejandra Navarro-Martínez, Marta Barón, Laura Navarro-Segura, Amaury Taboada-Rodríguez, Fulgencio Marín-Iniesta, Ginés Benito Martínez-Hernández

**Affiliations:** 1 Food Safety and Refrigeration Engineering Group, Department of Agricultural Engineering, Universidad Politécnica de Cartagena, Paseo Alfonso XIII 48, 30203 Cartagena, Spain; may.ros@upct.es (M.R.-C.); alejandra.navarro@upct.es (A.N.-M.); marta.baron@edu.upct.es (M.B.); laura.navarro@upct.es (L.N.-S.); 2 Group of Research Food Biotechnology-BTA, Department of Food Science, Nutrition and Bromatology, University of Murcia, Campus de Espinardo, 30100 Murcia, Spain; ataboada@um.es (A.T.-R.); fmarin@um.es (F.M.-I.); 3 Biotechnological Processes Technology and Engineering Lab, Instituto de Biotecnología Vegetal, Universidad Politécnica de Cartagena, Edif I+D+I, Campus Muralla del Mar, 30202 Cartagena, Spain

**Keywords:** EOs-MAP packaging, eugenol, polyphenoloxidase, phenyl ammonia lyase, quality, phenolic compounds

## Abstract

The use of vapour of essential oils (EOs) through an innovative pilot-plant packaging device was studied to preserve the quality of sliced mushrooms during storage. A mix of EOs (eugenol, bergamot EO, and grapefruit EO) was vaporized (100 and 125 µL L^−1^) and applied during packaging of sliced mushrooms under modified atmosphere packaging (MAP); then, the product quality was studied during cold storage up to 12 days. The highest colour changes of EOs125 samples, which were observed in the mushroom stipe, were not observed with the EOs100 atmosphere. Thus, the high polyphenoloxidase activity observed in untreated samples after 5–7 days was highly controlled with the vapour EOs atmospheres. Furthermore, the visual appearance scores of EOs100 samples were still over the limit of usability, while untreated samples were already below this threshold after 5 days of storage. A strong bacteriostatic effect was achieved with vapour EOs, reducing the *Pseudomonas* spp. (the main microbial genus in cultivated mushrooms) growth by ≈1.7 log CFU g^−1^, regardless of the EOs dose, after 12 days. The activity of phenyl ammonia lyase was also reduced up to ≈0.4 enzymatic units with the EOs100 treatment. Conclusively, packaging of sliced mushrooms under an atmosphere enriched with 100 µL L^−1^ EOs vapour highly controlled the quality loss of sliced mushrooms owing to their enzymatic inhibition and high bacteriostatic effect.

## 1. Introduction

Mushrooms of the *Agaricus* genera (primarily *A. bisporus*) constitute approximately 30% of the world’s cultivated mushrooms [1], with the major production located in China followed by the European Union (EU) (Poland is the major EU producer followed by The Netherlands and Spain) [2]. Only about 45% of total mushroom production is related to fresh consumption. Furthermore, the sliced mushrooms market has highly increased in the last decade owing to their convenience, although their shelf life is very short (a few hours) if appropriate postharvest techniques are not used.

The shelf life of white mushroom (also known as button mushroom) is about 1–3 days at ambient temperature (20–25 °C), or about 5–6 days when stored at 0–2 °C (reviewed by [3]). The short shelf life of mushrooms, and fungi in general, is mainly because they have no cuticle to protect them from physical or microbial attack and water loss [4]. Furthermore, their thin and porous epidermal structure leads to high respiration rates (132–158 and 20–35 mL CO_2_ kg^−1^ h^−1^ at 20 °C and 5 °C, respectively) [5,6]. Furthermore, mushroom slicing enhances microbial spoilage as it prompts an increment in the respiration rate and increases the surface area susceptible to dehydration, browning, and microbial growth.

The main quality changes, which highly limit mushroom shelf life, are discolouration (darkening or browning), moisture loss, texture softening, microbial spoilage, and flavour loss. Browning is mainly owed to enzymatic activity, principally by polyphenoloxidases (PPO) and bacterial blotch. Mushrooms have a high content of phenolic compounds [7], which, apart from being responsible for the high antioxidant activity of mushrooms, are the substrates for the browning reactions catalysed by the PPO. After harvesting, the typical firm texture (easily sheared and chewed) of fresh mushrooms becomes spongy and tough to chew and shear during their reduced postharvest life [8]. In particular, softening paralleled expansion of intercellular space at the pilei surface, hyphae shrinkage, central vacuole disruption, and loss of proteins and polysaccharides, while toughening was associated with increased chitin content [8]. Water loss of mushrooms during postharvest life is highly prevented when they are packaged with films with low water vapour permeability, which is of high importance in sliced mushrooms owing to their high water loss rates [9]. Nevertheless, special attention may be paid as such a high moisture content inside the product package may also encourage microbial growth. Cultivated mushrooms have high initial microbial loads (≈6–7 log CFU g^−1^), with *Pseudomonas* spp. being the main microbial group, which are even enhanced during mushroom slicing [10]. In accordance, several postharvest techniques like modified atmosphere packing (MAP), washing with antimicrobial agents (ascorbic acid, citric acid, hydrogen peroxide, and so on), irradiation, pulsed electric field, edible coatings, ozone, and electrolyzed water have been studied to extend the shelf life of produce like fresh mushrooms, controlling microbial growth, enzymatic activity, and other physiological processes (reviewed by [3,11,12]). Nevertheless, MAP supplementation with EOs vapour has not been studied yet in mushrooms.

Plant essential oils (EOs) have been conventionally considered as excellent postharvest treatments owing to their high antimicrobial properties. Furthermore, EOs mixes including major EOs components (e.g., eugenol) together with the EO have shown a synergistic effect with higher antimicrobial activity [13]. Furthermore, EOs are considered strong antioxidants, which are also able to inhibit the activity of quality-degrading enzymes like PPO or those involved in the phenolic biosynthetic pathway like phenyl ammonia lyase (PAL) [14,15]. Nevertheless, the effective EO doses determined with in vitro studies need to be increased under in vivo conditions, which may lead to the consumer rejection owing to EO-related off-flavours [1,2]. Furthermore, Eos’ solubility in water-based washing treatments is very low, with other techniques like nanoemulsions, encapsulation within cyclodextrins, and so on being needed.

Treatments with vapour EOs may overcome those drawbacks as they allow a rapid vaporization phase and homogeneous distribution of the antimicrobial EO vapours on the product surface [16,17]. EOs fumigation treatment (prior to packaging) of button and shitake mushrooms with vapour EOs and EOs components (cinnamaldehyde, clove EOs, and thyme EOs) reduced browning, decreased microbial load, and enhanced the contents of antioxidant compounds (i.e., phenolic compounds) and the activities of antioxidant enzymes [18,19]. Nevertheless, EOs fumigation prior to packaging may have a rebound effect as the surviving microflora to the EOs fumigation may display high growth during the product storage. Such a problem may be solved using alternative techniques like MAP supplemented with EOs vapour, which was proposed by our research group with our patented packing device of MAP with vapour EOs. We have observed excellent results in several plant and meat products (unpublished data) and fish fillets [20]. Nevertheless, MAP with vapour EOs has not been studied yet in mushrooms.

This work aimed to study the effect of MAP with vapour EOs (mix of eugenol, bergamot EO, and grapefruit EO) on the quality (texture, colour, microbial, sensory, pH, bioactive compounds, and the activity of relevant enzymes (i.e., PPO and PAL)) of sliced mushrooms (*A. bisporus*) stored at 4 °C up to 14 days. The hypothesis of this study was that the vapour EOs may highly control the activity of quality degrading enzymes (e.g., PPO versus colour) and exert a bacteriostatic effect, leading to better sensory quality and the extension of the product shelf life.

## 2. Materials and Methods

### 2.1. Materials

Mushrooms (*Agaricus bisporus* cv. Blanchocamp BL-40) were supplied by the company Champinter Soc. Coop. (Villamalea, Albacete, Spain) in January 2021. The mushrooms were grown at an industrial level using compost substrate under controlled conditions from mushroom mycelium.

In the company, mushrooms were manually harvested obtaining mushrooms with stipes of 3–5 cm long. Mushrooms were sliced (≈3 mm thickness) using an industrial slicing machine and then washed with sodium ascorbate solution (3%). Mushrooms slices were then packed within polystyrene trays, film-wrapped, and transported (at 4 °C in isothermal boxes for 2.5 h) to the pilot plant of the Agricultural Engineering Department of the Universidad Politécnica de Cartagena, where the packaging treatments with vapour EOs were conducted as described below.

Eugenol, bergamot EO, and grapefruit EO were supplied by Lluch Essence S.L. (Barcelona, Spain). Eugenol had a purity of 99%. The composition of eugenol, bergamot EO, and grapefruit EO mix is reported in point 2.2.

### 2.2. Packaging Treatments with Vapour Essential Oils

Packaging of sliced mushrooms with vapour EOs was conducted using a patented [21] device by our research group, which is able to package with vapour EOs through their vaporization under vacuum conditions (5–10 hPa). Previously, 150 g of sliced mushrooms was placed in a polypropylene basket (0.5 L capacity, which was then automatically sealed by the device using Cryovac^®^ EOP616B film (Cryovac; Fuenlabrada, Spain). This film (39 µm thickness) had gas/water transmission rates of O_2_, 7.00 cm^3^ m^−2^ day^−1^ atm^−1^; CO_2_, 25.00 cm^3^ m^−2^ day^−1^ atm^−1^; N_2_, 0.50 cm^3^ m^−2^ day^−1^ atm^−1^; and water, 10.00 g m^−2^ day^−1^. Sealing was done by the device with synthetic air atmosphere (Praxair; Murcia, Spain) supplemented with different doses (100 and 125 µL L^−1^) of the vapour EOs mix. The vapour EOs mix selected was eugenol/bergamot EO/grapefruit EO (60:20:20, volume, *v*:*v*:*v*) based on our preliminary in vitro experiments about the high antimicrobial effect of such an EOs mix against natural microflora isolated from sliced mushrooms. The used doses were selected based on our preliminary experiments with mushrooms slices in which the maximum EOs mix dose that did not transfer off-flavours/odours to the packaged product was 125 µL L^−1^ (data not published). A control treatment (CTRL) without addition of EOs was also conducted.

Three replicates (biological replicates) of one tray per treatment and storage time (processing day and after 2, 5, 7, 9 and 12 days) were prepared and stored in dark conditions in a cold room at 4 °C.

### 2.3. Gas Composition of Packages throughout Storage

The modified atmosphere generated (O_2_ consumption and CO_2_ generation until reaching the gas equilibrium) inside the packages was monitored during storage with a portable O_2_/CO_2_ analyser (CheckPoint PBI DANSENSOR, Ringsted, Denmark).

### 2.4. Physicochemical Analyses

Mushroom slices were blended (model MX2050; Braun, Marktheidenfeld, Germany), and the obtained blend was pressed throughout a four-layer cheesecloth to obtain some juice drops. Soluble solids content of the obtained juice drops was determined with a digital handheld refractometer (model N1; Atago, Tokyo, Japan) at 20 °C and expressed as Brix. For pH, 30 g of mushroom slices were blended with 20 mL of distilled water and the pH of the obtained blend was measured with a pH meter (Basic20; Crison, Alella, Spain).

The colour of samples was determined using a colourimeter (Chroma Meter CR-400, Konica Minolta, Tokyo, Japan) at illuminant D65 and 2° observer, with a viewing aperture of 8 mm. Three measurements were made per each sample, which were automatically averaged by the device.

Texture was determined with an Instron Universal Testing Machine (Ibertest eLib-5-W, Madrid, Spain) equipped with a Kramer standard shear cell. For each analysis, the Kramer cell was loaded with two layers of mushroom slices (four mushroom slices per each layer). The maximum shear force required to cross the two layers of mushroom slices was measured at a speed of 50 mm min^−1^ [9]. Three sets of measurements (three technical replicates) were made per each of the three biological replicates.

### 2.5. Sensory Analyses

Sensory analyses were performed according to international standards [22]. Sensory tests were conducted in a standard room [23] equipped with ten individual taste booths. The panel consisted of six assessors (four women and two men, aged 24–63 years old) who were familiarised with the product and scoring methods by demonstration exercises involving examination of packs at different levels of deterioration and agreeing appropriate scores. The quality attributes scored were overall appearance/visual quality, aroma, texture, and colour, as previously described [24]. The overall appearance/visual quality of the mushroom slices was evaluated using a nine-point numerical rating scale, where a score of 9 indicated the sample was excellent, 7 = very good, 5 = good and at the limit of marketability, 3 = fair and at the limit of usability, and 1 = poor and inedible. Aroma was determined after breaking the mushroom slices between the thumb and index finger and evaluating on a scale of 9–1, where 9 = full typical aroma or flavour, 7 = moderately full, 5 = moderate, 3 = poor, and 1 = none. Texture was evaluated when the mushroom was pressed between the thumb and index finger, on a scale of 9–1, where 9 = very firm and turgid, 7 = firm, 5 = moderately firm, 3 = soft, and 1 = very soft. Off-colours (browning) was evaluated based on a scale of 9–1, where 9 = no browning, 7 = slight to moderate, 5 = moderate, 3 = moderate to severe, and 1 = severe.

### 2.6. Microbial Analyses

Mesophiles, psychrophiles, enterobacteria, and *Pseudomonas* spp. microorganisms were analysed. Briefly, 15 g of samples were mixed with 150 mL of buffered peptone water and then homogenised for 1 min using a stomacher (Colwort Stomacher 400 Lab, Seward Medical; London, UK). Viable counts were based on counts by 10-fold serial dilutions in buffered peptone water. Then, aliquots (1 mL) of the microbial dilutions were pour-plated into Plate Count Agar, Violet Red Bile Dextrose Agar, and Cetrimide Agar for mesophiles/psychrophiles, enterobacteria, and *Pseudomonas* spp., respectively. All microbial media were purchased from Scharlau (Barcelona, Spain). Mesophiles, psychrophiles, enterobacteria, and *Pseudomonas* spp. were incubated at 31 °C (48 h), 4 °C (7 days), 37 °C (24 h), and 37 °C (48 h), respectively. The results were expressed as log colony forming units (CFU) g^−1^. Each of the three biological replicates was analysed in duplicate (technical replicate).

### 2.7. Enzymatic Activity

#### 2.7.1. Polyphenoloxidase (PPO)

The PPO activity was analysed based on previous literature [25,26], but with modifications. For enzyme extraction, 2.5 g of ground frozen sample (plus 75 mg of polyvinylpolypyrrolidone) was added to 7.5 mL of 100 mM potassium phosphate buffer (including 1% Triton X-100 and 1 M NaCl; pH 6.5) and homogenised (Ultraturrax; low speed) for 10 s at 2 °C. Samples were then centrifuged (14,000× *g*, 30 min, 4 °C) and the supernatant was used as the enzyme extract. First, 250 µL of 50 mM potassium phosphate buffer (pH 6.5) and 20 µL of enzyme extract were placed in a flat-bottom 96-well microplate (UV-STAR, Greiner Bio-One, Frickenhausen, Germany). Then, the reaction was initiated by adding 30 µL of a mix (freshly prepared before the assay) of 5 mM l-3,4-dihydroxyphenylalanine, 0.065 mM ethylenediaminetetraacetic acid, and 2.1 mM ascorbic acid (sodium salt) at 1:1:1 (*v:v:v*). PPO reaction (at 30 °C) was monitored for 10 min (every 1 min) at 265 nm in a microplate reader (Infinite M200; Tecan, Männedorf, Switzerland). PPO activity was calculated as the slope in the linear part of the activity curve obtained. One PPO activity unit (U) is defined as the decrease in absorbance of 0.001 units at 265 nm.

#### 2.7.2. Phenyl Ammonia Lyase (PAL)

The PAL extraction was conducted as previously described [27], but with slight modifications. For enzyme extraction, 2.5 g of ground frozen sample (plus 75 mg of polyvinylpolypyrrolidone) was homogenized with 5.5 mL of 0.05 M cold borate buffer (pH 8.5) containing 400 µL L^−1^ 2-mercaptoethanol. Homogenates were filtered through four-layer cheesecloth and then centrifuged at 10,000× *g* at 2 °C for 20 min. The supernatants were collected and used as PAL extracts. Two sets of UV-well plates containing 270 µL of PAL extract were prepared for every sample and preincubated at 40 °C for 5 min. Afterwards, 30 µL of either water (blank) or 100 mM L-phenylalanine substrate solution (freshly prepared before assay) was added to each of the wells for every sample set. The absorbances of the sample sets were measured at 290 nm at time 0 and after 1 h of incubation at 40 °C. The PAL activity was calculated as µmol of *t*-cinnamic acid synthesized kg^−1^ fresh weight (fw) h^−1^.

### 2.8. Total Phenolic Content

A single extract was prepared for total phenolic content (TPC) and total antioxidant capacity (TAC). For this, 2.5 g of frozen ground sample was homogenised (Ultraturrax; 10 s) in 10 mL of 80% MeOH (including 2 mM NaF). Extraction was followed by incubation (1 h, 5 °C) on an orbital shaker (Stuart SSL1; Stone, UK) at 60 cycles min^–1^. Samples were then centrifuged (14,000× *g*, 10 min, 4 °C) and supernatants were used as total phenolic/TAC extracts.

The Folin–Ciocalteu reagent method was used to analyse the TPC as previously described [28]. Briefly, 19 µL of the previous extract was placed on a flat-bottom PS 96-well plate and 29 µL of 1 N Folin–Ciocalteu reagent was added. The obtained mixture was incubated for 3 min at room temperature in darkness. After incubation, 192 µL of a solution containing 38 mM Na_2_CO_3_ and 500 mM NaOH was added and the reaction was carried out for 2 h at room temperature in darkness. Then, absorbance was measured at 750 nm using the microplate reader. TPC was expressed as gallic acid equivalents (GAE) in g kg^–1^ (fresh weight basis). Each of the three replicates was analysed in triplicate.

### 2.9. Total Antioxidant Capacity

The TAC was determined using the free radical 2.2-Diphenyl-l-pict3,1hydrazyl (DPPH●) [29]. TAC determinations of extracts were conducted as previously described [28]. Briefly, a solution of 0.7 mM DPPH was prepared in methanol 2 h before the assay and adjusted to 1.10 ± 0.02 nm immediately before use. A 21 µL aliquot of the extract was placed on a flat–bottom PS 96-well plate and 194 µL of the adjusted DPPH solution was added. The reaction was carried out for 25 min at room temperature in darkness and the absorbance at 515 nm was measured using the microplate reader. TAC was expressed as equivalents of TROLOX (6-hydroxy-2,5,7,8-tetramethylchroman-2-carboxylic acid) in µmol/kg (fresh weight basis). Each of the three replicates was analysed in duplicate.

### 2.10. Statistical Analyses

Statistical analysis was performed using the SPSS software (v.19 IBM, New York, NY, USA). Pairwise comparison was done using the independent samples *t*-test. Multiple groups were compared using analysis of variance (ANOVA), followed by the Tukey’s honest significant difference *post hoc* test. In every case, statistical significance was assessed at *p* = 0.05.

## 3. Results and Discussion

### 3.1. Gas Composition

The steady-state gas equilibrium of the generated modified atmosphere (O_2_ uptake and CO_2_ evolution) was reached early at day 2. The reached equilibrium was 7.8 ± 2.1 O_2_ and 10.6 ± 1.8 CO_2_ (mean values from day 5 to day 12), without large differences among the studied EOs’ MAP treatments. This fast generation of the steady-state gas equilibrium has been also observed in sliced mushrooms under MAP at 4 °C [9]. This may be explained by the high respiration rate of mushrooms (≈20–35 mL kg^−1^ h^−1^ at 4 °C), which is even increased when they are sliced [5,6].

Sliced mushrooms packaged under MAP (previously sanitized with citric acid) reached a shelf life of 12–17 days at 4–5 °C [9,10]. The hereby reached steady-state gas equilibrium agrees with the recommended MAP equilibrium ranges reported in the literature [30]. O_2_ atmospheres of 8–10% highly controlled microbial growth, mainly *Pseudomonas* spp. with 1.3–1.7 log cycles lower counts after 13 days at 3 °C [31]. Furthermore, higher browning has also been observed in mushrooms packaged under MAP with 10–12% CO_2_ [4,31]. On the contrary, CO_2_ and O_2_ levels of 3.8–6.9% and 2–6.3%, respectively, were not enough to reach high microbial reductions (<0.8 log cycles of reduction) in sliced mushrooms [10]. Thus, the achieved CO_2_ and O_2_ levels of the steady-state gas equilibrium are in accordance with the optimum range recommended for mushrooms [30].

### 3.2. pH

Mushrooms slices had an initial pH of 6.75 (Table 1). The time factor was significant for pH (Table 1), with an observed pH increment of 0.4–0.45 pH units from day 0 to day 2. This pH increment trend was observed during the rest of the storage period, but to a lower degree (changes < 0.3 pH units from day 2 to day 12). Organic acids of horticultural products, and mushrooms in particular (from higher to lower content: citric acid > malic acid > oxalic acid > fumaric acid; [32]), are used as energy sources during metabolic processes of plant cells. In particular, the initial plant stress at harvest leads to a high plant cell response, which demands high energy obtained from plant metabolites like organic acids leading to the observed pH increment. Once the product metabolism is stabilized to the refrigeration storage temperature, such high energy demand is reduced [30].

The treatment factor, as well as the time × treatment interaction, were significant for pH (Table 1). Thus, sliced mushrooms stored under the EOs125 atmosphere registered the lowest pH changes throughout storage with pH increments of 0.35 and 0.48 after 9 and 12 days, respectively. However, the lowest EOs dose (EOs100) was not enough to control the metabolic processes that highly result in pH changes, with EOs100 samples showing similar pH increments after 9–12 days (0.6–0.7 pH units) to CTRL samples. The observed protective effect of EOs atmospheres to minimize the product metabolism, as observed from the lower pH changes, has been observed by our group in different horticultural produce, except for mushrooms, which are firstly reported in this study.

### 3.3. Texture

Sliced mushrooms showed an initial shear force of 366 N (Table 1). Time factor, as well as EOs dose and the time × dose interaction, were significant (*p* < 0.05) for the shear force. Thus, shear force highly increased (≈2-fold) in the first two days of storage. The shear force increment has been associated with mushroom toughening, while mushroom firmness (softening; measured by puncturing force) is reduced [8,9,10]. The cell wall of mushrooms mainly consists of glucans, chitin, and protein [33]. Different from other fruit and vegetables, mushrooms lack a pectin structure [3]. Chitin is the skeletal component comprising 30–50% of the cell walls of mushroom, being an integral component of the cell wall and contributing to the structural rigidity and osmotic integrity [34]. Toughening of mushroom during storage has been associated with an increment of the chitin content while softening paralleled expansion of the intercellular space at the pileus surface, hyphae shrinkage, central vacuole disruption, and loss of proteins and polysaccharides [8]. Disruption of the plant cell wall, such as that occurred during senescence processes of mushrooms, results in compensatory alterations in the cell wall, for example, enhancing the synthesis of the cell wall polymers (through activation of enzymes like glucan synthase and chitin synthase) in an attempt to maintain cellular integrity [33,35], which explains the findings found here.

Different shear force patterns were observed during mushroom storage depending on the EOs’ atmosphere treatment (Table 1). The shear force of CTRL samples remained unchanged (*p* > 0.05) from day 2 to day 7, and then started to decrease. This shear force decrease at the end of the storage of mushroom slices has been previously observed [9,10], and it has been related to tissue degradation owing to senescence processes and microbial spoilage of samples. Contrary to EOs125 samples, EOs100 did not increase (*p* > 0.05) the toughening of samples from day 2 until the end of storage (Table 1). On the other side, the shear force of EOs125 samples showed an increment at day 9, followed by a decreasing trend at day 12 (similar to CTRL samples). The observed increment of EOs125 samples at day 9 may be owing to a chitin and/or glucan biosynthesis through the activation of chitin and glucan synthases as an abiotic stress response to that high EOs dose. Interestingly, the increment of lignin, another key structural compound of mushroom cell walls, has been previously hypothesised as a response to several abiotic stresses in shredded carrots [36]. Nevertheless, texture measurement is very complex, with several parameters like gumminess, hardness, and fracturability playing a high importance for mushroom texture [8]. Thus, texture measurements of mushroom slices need also to be contrasted with texture quality scored in sensory analyses.

### 3.4. Colour and Polyphenol Oxidase Activity

Colour is the quality attribute of mushrooms that highly affects consumers’ purchasing decision, who expect a product with full white colour without darkened/brown areas. In that sense, luminosity (L*) values of caps and stipes were 92.1 and 89.2, respectively (Table 1). The time factor was significant (*p* < 0.05) for L* values. Thus, an L* decrease of samples was observed during storage as a consequence of mushroom browning.

**Figure 1:**
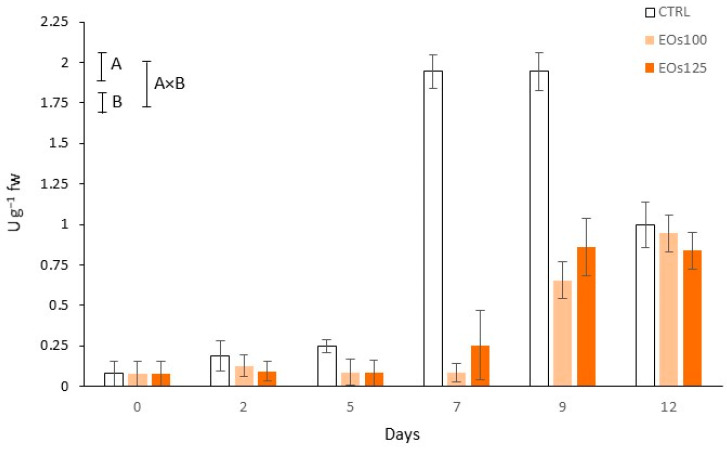
Polyphenoloxidase activity of sliced mushroom during cold storage (4 °C) under modified atmosphere packaging supplemented with vapour essential oils (100 and 125 µL L^−1^ EOs) (mean ± standard deviation). Least significant differences are represented as bars, with A and B letters for the treatment and storage time factors, respectively.

PPO activity, tyrosinase in particular for mushrooms, is regarded as the main reason for mushroom browning during post-harvest life [3]. In the PPO-catalysed reactions, phenolic compounds are oxidised into quinones, which are then converted to melanin, producing the browning appearance of mushrooms. In accordance, initial PPO activity (0.08 U g^−1^) of samples (Figure 1) increased through storage (time factor was significant for PPO activity; *p* < 0.05), reaching enzymatic increments up to 10–13-fold after 12 days of storage.

**Figure 2:**
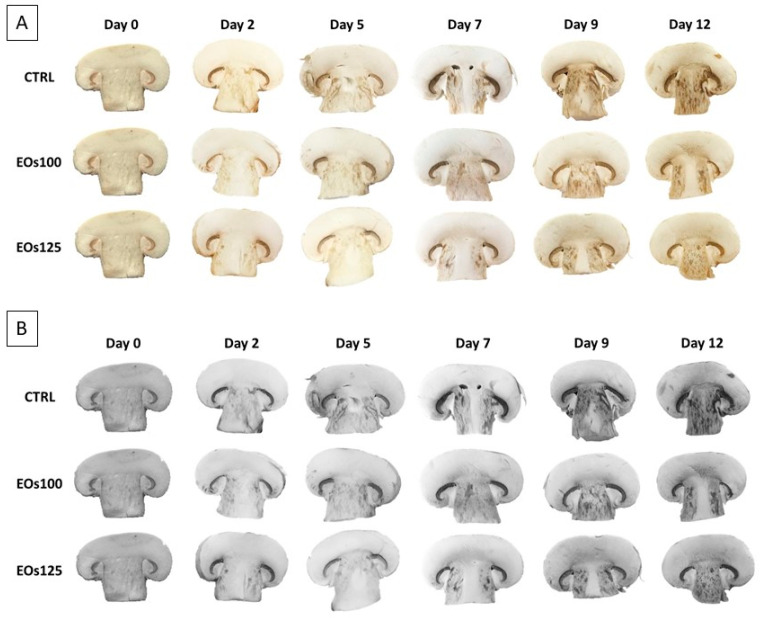
Sliced mushroom during cold storage (4 °C) under modified atmosphere packaging supplemented with vapour essential oils (100 and 125 µL L^−1^ EOs). (**A**) Original RGB image; (**B**) BW image.

Browning in stipes was higher than in caps (Figure 2) with L* reduction slopes (L* value reduction per day) of 0.87–1.00 and 1.10–1.15, respectively. The higher browning in the stipes may be owing to higher phenolic content and higher PPO activity in the stipe compared with the cap, as widely reported in the literature [7,37,38]. L* values of caps were above 80 during all storage periods, except L* values of EOs125 that were slightly lower than 80 on day 12. These L* values may be regarded as acceptable in accordance with published criteria [9,39], which considered L* values above 80 in mushroom caps to be acceptable. In that sense, stipe browning was the major off-colour aspect that panellists found according to their comments (external to sensory scores).

EOs dose factor was significant (*p* < 0.05) for both L* values and PPO activity (Table 1 and Figure 1). In general, samples under EOs atmospheres showed slightly lower L* values, without differences (*p* > 0.05) among EOs doses. Nevertheless, the lowest dose (EOs100) minimized such an effect of EOs atmospheres on mushroom whiteness, showing similar L* values to CTRL samples after 12 days. Interestingly, a high PPO activation (≈8-fold) was observed in CTRL samples from day 5 to day 7, which was not detected in samples under EOs atmospheres. The inhibition of PPO activity by other EOs has already been observed in mushrooms and other plant products [14,15,18,40]. Thus, a competitive inhibition of EOs has been demonstrated within the active sites of PPO in fresh-cut lettuce [14]. Therefore, panellists observed a high browning of CTRL mushroom stipes after 7 days, with browning scores of CTRL samples below the limit of usability from day 7 (see Section 3.5). However, such high browning in mushroom stipes scored in the sensory analyses was not observed in L* data. This finding may be explained by the heterogeneity of browned areas in the stipes (see Figure 2), which led to the high variability of the colourimeter measurements among samples, as also observed from the high standard deviations).

Conclusively, although no large differences among cap L* values of the different treatments were found, EOs atmospheres highly inhibited the high PPO activation observed in untreated samples after 7 days of storage. Although a high variability among browning (L* values) areas in stipes was observed, the intense browning in CTRL stipes was clearly appreciated in the sensory analyses. On the contrary, EOs100 samples were the only samples above the limit of usability after 12 days, as shown in the following section.

### 3.5. Sensory Analyses

**Figure 3:**
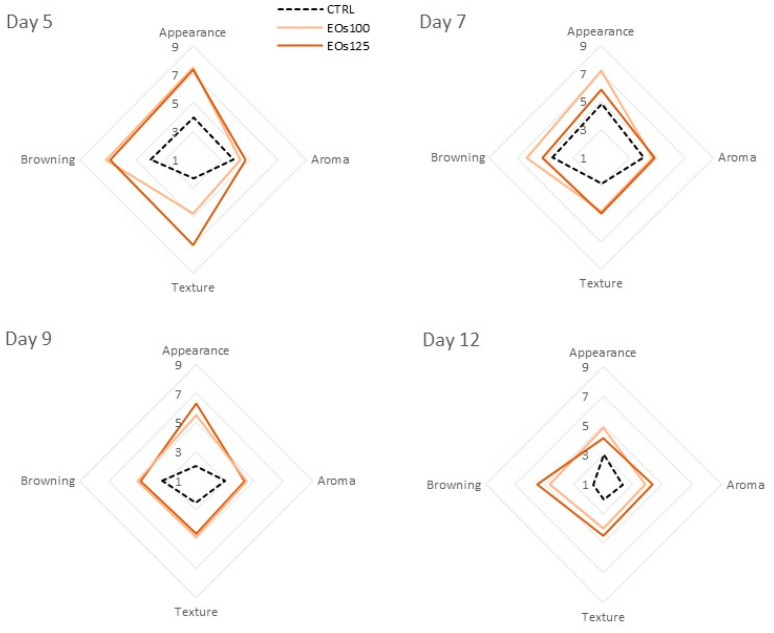
Sensory analyses of sliced mushroom during cold storage (4 °C) under modified atmosphere packaging supplemented with vapour essential oils (100 and 125 µL L^−1^ EOs).

The visual appearance of mushrooms is the most important quality attribute in the purchase decision. In particular, browning is the main colour degradation in mushrooms. Browning of samples was predominantly located in the stipe section, which started from the perimeter and advanced to the inner stipe throughout storage (see Figure 2). Lower browning was observed in the cap section, which agrees with L* data. On the contrary, mushroom browning is mainly located in the cap surface (owing to the higher phenolic contents and PPO activity on the cap surface tissues), which is less appreciated in sliced mushrooms, as hereby commented. A high stipe browning was already perceived at day 5 in CTRL samples (Figure 2) with a score of 4.0 (Figure 3), while browning of samples under EOs atmospheres was scored with 6.8–7.2 at day 5. Although stipe browning was intensified throughout storage in all samples, browning of EOs125 samples remained above (score 5.5) the limit of usability after 12 days, and EOs100 was also almost on the limit of usability (score 4.7). The visual appearance of mushrooms is highly affected by enzymatic browning and bacterial blotch, which is mostly located on the cap surface (not well appreciated in sliced mushrooms). In that sense, visual appearance scores followed the same trend as browning scores. Water loss of samples may also lead to water condensations and mushroom shrinkage, which may highly deteriorate the visual appearance of samples. Nevertheless, panellists did not appreciate remarkable water condensations within packages, which agrees with the very low water losses of samples after 12 days (<1%). This low water loss is far from the reported threshold of 5%, at which mushrooms are regarded as perished [41]. This low water loss may be explained by the limitation of the product transpiration through the reduction of its respiration rate as a consequence of the reached modified atmosphere (reduced O_2_ and increased CO_2_).

Regarding aroma, CTRL samples showed lower aroma scores than EOs samples (Figure 3). In particular, the aroma of CTRL samples was scored with approximately 3 after 9–12 days, while samples under EOs atmospheres showed scores close to 5. Aroma is an important quality parameter in mushrooms, with earthy, hay, soybean, potato, and woody nuances being the main components of the aroma quality in mushrooms [42]. The aroma quality is even more important in sliced mushrooms because its intensity is higher owing to the leached aroma volatiles from disrupted cells. As observed, packaging of sliced mushrooms under EOs atmospheres, regardless of the dose, preserved its aroma quality during storage. No off-odours related to EOs were perceived for any of the EOs treatments.

As previously discussed, other texture parameters than the measured shear force contribute to the mushroom texture scored by panellists. In that sense, texture of CTRL samples was affected early at day 5, with scores below the limit of usability (Figure 3). On day 9, texture scores of samples under EOs were still close to the limit of usability (≈4.8), while the texture score for CTRL samples was 2.5. As observed, different conclusions may be obtained when measuring the shear force of samples or when texture is scored with sensory analyses by pressing the mushroom slices between the fingers. Thus, the sensory texture data, which indicate a more realistic approximation of the consumer appreciation of mushroom texture, show that EOs atmospheres (without significant differences among both EOs doses) better preserved the sensory texture of sliced mushrooms for almost 9 days of storage.

### 3.6. Microbial Quality

The initial total aerobic count was 4.9 log CFU g^−1^ (Table 2). High total aerobic counts have been widely reported (reviewed by [10]) in cultivated mushrooms ranging from 6.2 to 7.2 log CFU g^−1^. This high bacterial load in fresh mushrooms is a major aspect that significantly diminishes mushroom quality by causing a brown, blotchy appearance [4]. The lower initial counts of our samples are explained as sliced mushrooms received from the company were already sanitized, as detailed in the Material and Methods section.

The major bacterial group present in mushrooms is identified as fluorescent *Pseudomonas* spp. owing to contamination of the product from compost [4,10,18]. Sliced mushrooms showed an initial *Pseudomonas* spp. load of 2.3 log CFU g^−1^ (Table 2). The time factor was significant for *Pseudomonas* spp. counts (Table 2). Initial microbial growth of 1.6–2.5 log units was observed in the first two days of storage, with the microbial growth rates being reduced in the subsequent storage days. The reduction of microbial growth starting from day 2 may be owing to the reached increased CO_2_ and reduced O_2_ concentrations of the gas steady-state equilibrium, which minimized the microbial growth, as observed. Attending to EOs treatments, no high differences (<1 log units) were observed between EOs100 and EOs125 treatments in the first 9 days of storage, with loads below 6 log units. Nevertheless, the microbicidal effect of EOs atmosphere was observed from day 9. The highest EOs effect was observed at day 12, with counts 1.7 log units lower than CTRL samples, regardless of the EOs dose.

A similar microbicidal effect of EOs was observed for Enterobacteria, with 1.2–1.5 lower log units compared with CTRL samples at day 12, without differences (*p* > 0.05) among the EOs doses (Table 2). Psychrophilic loads showed a similar trend, although no significant differences were observed at day 12 among samples.

Conclusively, a high inhibitory action was observed with the studied EOs atmospheres against *Pseudomonas*, the most abundant microbial genus in cultivated mushrooms. Interestingly, such a microbicidal effect was better observed in the latter storage days, when it is crucial that applied treatments still retain their bacteriostatic effect. The higher microbial growth of untreated (CTRL) samples in these latter storage days allowed us to observe the bacteriostatic effect of EOs atmospheres.

### 3.7. Phenolic Compounds and Phenyl Ammonia Lyase Activity

**Figure 4:**
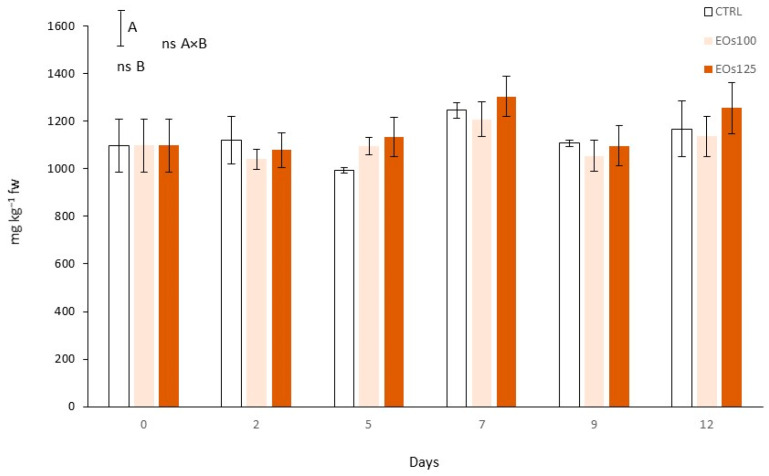
Total phenolic content of sliced mushroom during cold storage (4 °C) under modified atmosphere packaging supplemented with vapour essential oils (100 and 125 µL L^−1^ EOs) (mean ± standard deviation). Least significant differences are represented as bars, with A and B letters for the treatment and storage time factors, respectively. ns, not significant.

**Figure 5:**
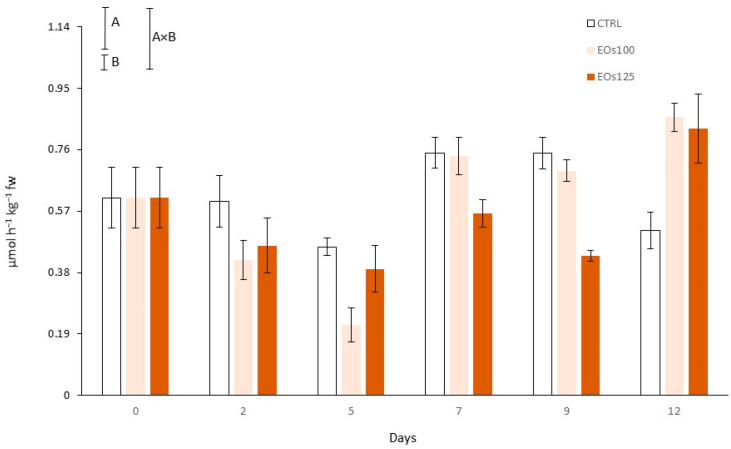
Phenyl ammonia lyase activity of sliced mushroom during cold storage (4 °C) under modified atmosphere packaging supplemented with vapour essential oils (100 and 125 µL L^−1^ EOs) (mean ± standard deviation). Least significant differences are represented as bars, with A and B letters for the treatment and storage time factors, respectively.

Mushroom slices had an initial TPC of 1097.8 mg kg^−1^ (Figure 4). Phenyl ammonia lyase is the key enzyme in the biosynthesis pathway of phenolic compounds [43,44]. The time factor was significant (*p* < 0.05) for both TPC and PAL activity (Figure 4 and Figure 5). EOs dose factor and the interaction time × EOs dose were also significant for PAL activity. The initial PAL activity (0.61 µmol h^−1^ kg^−1^) was reduced in the first days of storage, with EOs showing an inhibitory effect of PAL activity. Thus, PAL activity was reduced early under EOs atmospheres by 0.15–0.19 PAL units, regardless of the EOs’ dose (*p* > 0.05), compared with their respective initial values at day 0, while CTRL remained unchanged (*p* > 0.05). The PAL-inhibitory effect of EOs was more pronounced at day 5, with EOs100 showing the highest inhibition with a PAL activity 0.39 units lower than the initial values. The inhibitory effect of EOs against PAL activity has already been observed in other plant products, although the mechanism of such inhibitory effect is still unknown. A similar mechanism could be hypothesized as that demonstrated for PPO through competitive inhibition of EOs within the active sites of the enzyme [14].

TPC of EOs samples remained without high changes in the first 5 days, which is explained by the observed low PAL activity and lower consumption of phenolic compounds as substrates during the browning process. On the other side, the low TPC of CTRL at day 5, compared with EOs samples, may be owing to higher consumption as a substrate during mushroom browning. PAL activity of all samples increased by 2–3-fold from day 5 to day 7. EOs125 reduced PAL activity by 1.3–1.7-fold compared with CTRL samples at days 7–9. Nevertheless, the EOs100 dose was not enough to reach that PAL inactivation degree. Contrary to the high PAL differences between treatments, EOs dose factor was not significant for TPC, which could be masked by the phenolic consumption during browning processes.

### 3.8. Total Antioxidant Capacity

**Figure 6:**
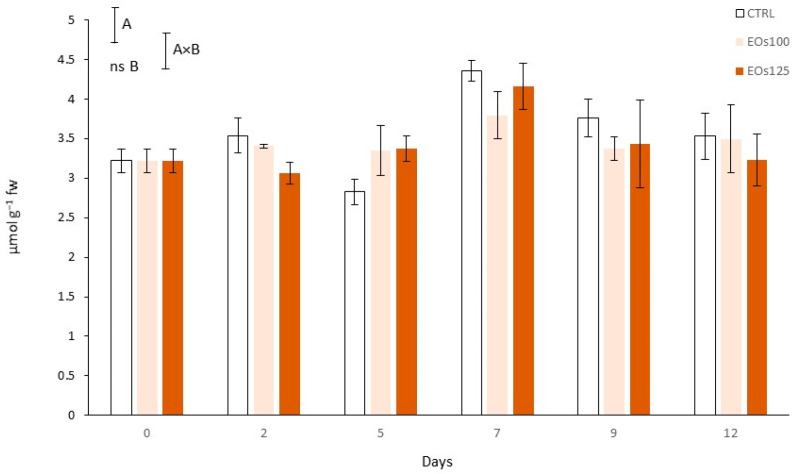
Total antioxidant capacity of sliced mushroom during cold storage (4 °C) under modified atmosphere packaging supplemented with vapour essential oils (100 and 125 µL L^−1^ EOs) (mean ± standard deviation). Least significant differences are represented as bars, with A and B letters for the treatment and storage time factors, respectively. ns, not significant.

An initial TAC of 3.22 µmol g^−1^ was observed at day 0 (Figure 6). A TAC increment (*p* < 0.05) was observed at day 7 with values 1.2–1.4-fold higher compared with their respective levels at day 0. This TAC increase at day 7 is in accordance with the observed PAL activity and TPC increments. This high TPC–TAC correlation may be explained because phenolic compounds have been reported as the major antioxidant compounds in mushrooms, which are mainly concentrated in the mushroom gills (3.6-fold higher contents than in caps and stipe) [7]. Thus, TPC was highly correlated with TAC in our study with a *R*^2^ = 0.7. In that sense, TAC decreased from day 7 to day 12, as similarly observed for TPC, without significant differences (*p* > 0.05) among treatments.

## 4. Conclusions

The application of vaporized essential oils within the modified atmosphere packaging is an innovative approach that may reduce the quality loss of sliced mushrooms during postharvest storage. The achieved essential oil atmosphere within packages reduced the polyphenoloxidase activity of sliced mushrooms, with a dose of 100 µL L^−1^ being enough to achieve such an inhibitory effect of this enzyme, with visual appearance scores above the limit of usability after 12 days. Similarly, vapour essential oils also showed an inhibitory effect of the phenyl ammonia lyase activity. A strong bacteriostatic effect was achieved with this innovative approach, reducing *Pseudomonas* spp. (the most important bacteria group in cultivated mushrooms) growth by approximately twofold after 12 days at 4 °C. Thus, although the overall quality of sliced mushrooms was highly maintained using an essential oils atmosphere of 100 µL L^−1^, further research must be conducted to better study the correlation of the observed toughening of samples with the changes of cell wall components. Furthermore, different mixes of essential oils would lead to better control of those aspects.

## Figures and Tables

**Table 1:** pH, firmness (shear force), and colour (L*) of sliced mushroom during cold storage (4 °C) under modified atmosphere packaging (MAP) supplemented with vapour essential oils (100 and 125 µL L^−1^ EOs) (mean ± standard deviation). Least significant differences are represented within parentheses.

Vapour EOs MAP	Storage Time (Days)	pH	L* (Cap)	L* (Stipe)	Shear Force (N)
Processing day		6.75 ± 0.05	92.1 ± 0.8	89.2 ± 0.6	366.2 ± 65.0
CTRL	2	7.20 ± 0.12	89.6 ± 1.3	88.4 ± 1.7	707.2 ± 75.8
	5	6.96 ± 0.20	89.2 ± 1.0	85.2 ± 1.7	598.3 ± 52.8
	7	6.95 ± 0.01	86.4 ± 3.0	81.0 ± 2.9	621.7 ± 76.6
	9	7.38 ± 0.14	83.6 ± 2.5	76.9 ± 1.9	548.3 ± 61.5
	12	7.41 ± 0.02	79.9 ± 2.0	75.0 ± 3.8	526.8 ± 76.9
EOs100	2	7.15 ± 0.02	87.9 ± 1.9	88.9 ± 1.5	784.9 ± 73.2
	5	6.94 ± 0.14	88.1 ± 4.3	85.0 ± 1.4	707.2 ± 56.7
	7	7.29 ± 0.09	84.2 ± 2.2	80.8 ± 3.7	803.1 ± 127.8
	9	7.47 ± 0.01	80.3 ± 1.9	76.6 ± 2.4	643.4 ± 61.2
	12	7.51 ± 0.01	79.5 ± 2.4	75.5 ± 5.0	752.5 ± 133.7
EOs125	2	7.13 ± 0.04	88.7 ± 1.4	87.0 ± 2.3	811.8 ± 85.9
	5	7.03 ± 0.28	87.6 ± 1.7	83.5 ± 2.3	786.6 ± 52.0
	7	7.39 ± 0.06	84.3 ± 1.5	79.6 ± 2.9	930.9 ± 142.1
	9	7.09 ± 0.04	81.0 ± 1.1	75.7 ± 4.2	835.0 ± 118.5
	12	7.23 ± 0.04	78.2 ± 1.4	74.5 ± 2.4	726.3 ± 182.1
Treatment (A)		(0.07) *	(1.1) ‡	(1.4) ‡	(109.1) ‡
Storage time (B)		(0.18) ‡	(1.6) ‡	(1.9) ‡	(154.3) ‡
A × B		(0.31) ‡	ns	ns	(267.3) ‡

ns: not significant (*p* > 0.05); * and ‡ significance for *p* ≤ 0.05 and 0.001, respectively.

**Table 2:** Microbial loads (log CFU g^−1^) of sliced mushrooms during cold storage (4 °C) under modified atmosphere packaging (MAP) supplemented with vapour essential oils (100 and 125 µL L^−1^ EOs) (mean ± standard deviation).

Vapour EOs MAP	Storage Time (Days)	*Pseudomonas*	Mesophilic	Psychrophilic	Enterobacteria
Processing day		2.29 ± 0.03	4.87 ± 0.07	3.24 ± 0.95	1.95 ± 0.15
CTRL	2	4.73 ± 0.06	6.67 ± 0.11	5.32 ± 0.07	3.96 ± 0.41
	5	5.84 ± 0.01	7.56 ± 0.04	7.45 ± 0.01	5.74 ± 0.24
	7	4.92 ± 0.11	7.81 ± 0.21	7.95 ± 0.17	6.06 ± 0.14
	9	5.72 ± 0.11	8.20 ± 0.06	8.21 ± 0.15	6.82 ± 0.24
	12	5.79 ± 0.54	9.00 ± 0.12	9.15 ± 0.13	7.41 ± 0.13
EOs100	2	4.32 ± 0.02	6.19 ± 0.02	4.90 ± 0.04	4.26 ± 0.21
	5	5.93 ± 0.25	7.44 ± 0.17	6.72 ± 0.14	5.54 ± 0.34
	7	5.71 ± 0.17	7.61 ± 0.28	7.60 ± 0.56	5.70 ± 0.29
	9	4.74 ± 0.49	8.10 ± 0.10	8.22 ± 0.20	6.32 ± 0.28
	12	4.09 ± 0.19	8.60 ± 0.09	8.91 ± 0.12	6.21 ± 0.38
EOs125	2	5.31 ± 0.73	6.58 ± 0.02	5.58 ± 0.20	4.99 ± 0.38
	5	5.74 ± 0.36	7.60 ± 0.14	7.57 ± 0.06	5.74 ± 0.09
	7	6.29 ± 0.25	7.82 ± 0.10	7.97 ± 0.18	5.67 ± 0.59
	9	5.75 ± 0.11	9.38 ± 0.09	9.00 ± 0.38	7.13 ± 0.59
	12	4.13 ± 0.30	8.87 ± 0.09	9.13 ± 0.08	5.87 ± 0.79
Treatment (A)		(0.46) ‡	(0.19) ‡	(0.42) ‡	(0.67) ‡
Storage time (B)		(0.65) ‡	(0.27) ‡	(0.59) ‡	(0.94) ‡
A × B		(1.12) ‡	(0.47) ‡	ns	(0.92) *

Least significant differences are represented within parentheses. ns: not significant (*p* > 0.05); * and ‡ significance for *p* ≤ 0.05 and 0.001, respectively.

## Data Availability

The data presented in this study are available on request from the corresponding author.
